# The Microbial Community Dynamics during the Vitex Honey Ripening Process in the Honeycomb

**DOI:** 10.3389/fmicb.2017.01649

**Published:** 2017-08-29

**Authors:** Yaqin Wen, Lin Wang, Yue Jin, Jinzhen Zhang, Lei Su, Xiaoling Zhang, Jinhui Zhou, Yi Li

**Affiliations:** ^1^Institute of Apicultural Research, Chinese Academy of Agricultural Sciences Beijing, China; ^2^Key Laboratory of Bee Products for Quality and Safety Control, Ministry of Agriculture Beijing, China; ^3^Laboratory of Risk Assessment for Quality and Safety of Bee Products, Ministry of Agriculture Beijing, China; ^4^Key Laboratory of Microbial Resources, Ministry of Agriculture, Institute of Agricultural Resources and Regional Planning, Chinese Academy of Agricultural Sciences Beijing, China; ^5^State Key Laboratory of Mycology, Institute of Microbiology, Chinese Academy of Sciences Beijing, China

**Keywords:** microorganism, community, honey ripening process, 16S RNA gene, ITS region

## Abstract

The bacterial and fungal communities of vitex honey were surveyed by sequencing the 16S rRNA gene and the internal transcribed spacer region of ribosomal DNA. Vitex honey samples were analyzed at different stage of ripening; the vitex flower was also analyzed, and the effect of the chemical composition in the experimental setup was assessed. The results confirmed the presence of dominant *Bacillus* spp. as the dominant bacterial in honey, and yeast related genera was the main fungal in the honey, respectively. *Lactococcus* and *Enterococcus* were detected for the first time in honey. The proportion of most of the fungal community decreased during the honey ripening process. Multivariate analyses also showed that the fungal community of 5, 10, and 15 days honey samples tended to cluster together and were completely separated from the 1 day honey sample. The change in the fungal community showed a correlation with the variation in the chemical components, such as moisture and phenolic compounds. Together, these results suggest that ripening of honey could change its microbial composition, and decrease the potential risk of microbiology.

## Introduction

Honey is a naturally sweet substance that is made when the nectar and sweet deposits from plants are gathered, modified and stored in the honeycomb by honeybees. The honey ripening process is the conversion of nectar to honey by honey bees (*Apis mellifera* L.) and involves the removal of water and the addition of a few enzymes that changes the major sugars in the nectar (Lichtenberg-Kraag, 2014). Generally, the conversion from nectar to honey takes 1–3 days (White, 1992; Ball, 2007). Bees continue to add nectar to individual honeycomb cells until the chamber is full, after which the bees cap the cell with newly produced beeswax (White, 1992; Ball, 2007). The capping process may require 7–9 days up to a month depending on the flowering phase, climate, bee colony and other factors. More than 180 different components can be detected in the honey (da Silva et al., 2016), with many of them generated or modified during the ripening process (Vyviurska et al., 2016).

The antimicrobial effects of honey (mainly antibiotic and antiseptic) have been shown in several studies. These effects are primarily due to the composition of honey, which consists of a high sugar and low water concentration with a low pH. Honey also contains bacterial inhibiting molecules, such as hydrogen peroxide produced by glucose oxidase; non-peroxide inhibins are additionally present, which are also known as phenolic components, aromatic acids, and other phytochemical compounds (Lee et al., 2008; Ceausi et al., 2009; Laallam et al., 2015). Consequently, honey can be expected to contain a small number and a limited variety of microorganisms.

Despite the presence of numerous inhibiting factors, some microorganisms can survive in honey. The previous researches focused on the detection of microorganisms in honey with a primary focus on (1) microorganisms that are commonly found in honey, such as certain yeasts and spore-forming bacteria; (2) microorganisms that indicate sanitary or commercial quality, such as coliforms or yeasts, as well as microorganisms that can cause illness under certain conditions (e.g., germination and growth in a non-heated-treated product) (Snowdon and Cliver, 1996). The microbial populations in French (Tysset and Rousseau, 1981), Italian (Piana et al., 1991), Sardinian (Farris et al., 1986; Sinacori et al., 2014) and Argentinean honeys (Iurlina and Fritz, 2005) were determined and characterized, and the results showed that the number of bacteria, molds and yeasts detected in honey were lower. The characterization of molds and yeasts of honey samples from Poland showed that the amount of fungi was less than 10^2^ CFU (Felsociova et al., 2012). Different botanical types of Polish honey have different levels of bacteria and low levels of yeasts and molds (Rozanska, 2011). Seven honey yeast species belonging to six different genera were identified in Portugal honey by using RFLP analysis of the ITS region (Carvalho et al., 2005). Bacteria, such as coliforms, enterococci, bacilli, as well as fungi belonging to the genera *Penicillium, Cladosporium* and *Alternaria* were monitored in Slovakian honeys (Kacániová et al., 2009). In addition, 13 bacteria, 5 yeasts and 17 filamentous fungi were isolated in honey by using a culture-dependent approach, with the species most frequently isolated being *Bacillus amyloliquefaciens, Zygosaccharomyces mellis*, and *Aspergillus niger* as representatives of the three microbial groups (Sinacori et al., 2014). Both in natural honey and in synthetic syrup, the bacterial population decreases over the course of the ripening process. *Lactobacillus* and *Gluconobacter* disappear after the minimum moisture content (∼18%) is reached, but the former does so sooner than the latter (Ruiz-Argueso and Rodriguez-Navarro, 1975).

The primary sources of the microbial community present in honey are pollen, the digestive tracts of honey-bees, and microorganisms normally present in dust, air, and flowers (Snowdon and Cliver, 1996; Kacániová et al., 2009). Cultivation and sequencing were used to explore bacterial communities in floral nectar, the honey bee alimentary tract, honey and packed pollen. A typical adult honey-bee gut is often colonized by the some reported probiotic bacteria *Lactobacillus, Bifidobacteria*, and *Bacillus* (Kacániová et al., 2009; Engel et al., 2012). Certain Yeasts were isolated from nectars, such as *Metschnikowia, Cryptococcus* (Lievens et al., 2015). *Metschnikowia* related species are typically isolated from flowers or fruits and transmitted to new niches by insects (Hong et al., 2001). These results revealed that many that are bacteria prevalent in honey and the bee foregut were also found in floral nectar, suggesting frequent horizontal transmission (Aizenberg-Gershtein et al., 2013; Anderson et al., 2013).

Recent many researches focused on the non-culture based investigations of the microbial diversity of foraging bee, bees captured from within the hive, bee gut, food stores and the pollination environment. Less attention has been paid to the microbial diversity of honey ripening process, and the flowers. The quality of honey depends not only on its physical and chemical properties but also on the microbiological aspects. Honey is directly eaten as food or used as a food ingredient, and its microbial load may be transferred to complex matrices, where some microorganisms may find optimal conditions in which to grow. The natural ripening of honey in the comb is a key point in the honey-making process. In the Chinese bee industry, some beekeepers harvest the raw honey every 2 or 3 days, resulting in a high moisture in raw honey and decreased antibacterial activity. Knowledge of the microbial composition and species abundance during the natural ripening process of honey may provide important insight for the correct management of this process.

In the study, honey samples at different ripening stages in hive were selected to investigate changes in bacterial and fungal composition and their diversity using culture-independent sequencing technology. In addition, honey chemical properties, like moisture and phenolic profile were analyzed for its correlation with the microbial community changes during ripening process. Our objectives were to determine (1) how the changes of honey bacterial and fungal communities vary with honey ripening process; and (2) to assess the potential risk of microbial pathogens in honey sample under different ripening stages.

## Materials and Methods

### Honey and Flower Samples

Vitex honey samples were harvested from a beekeeper in the Mentougou district in Beijing. Six beehives were positioned approximately 2 m far from the vitex bushes. The six beehives were positioned at same distance from the vitex bushes and randomly divided into three groups of biological duplicates. At the beginning of the experiment, two empty combs were placed in the honey hive. Honey samples from the two beehives in same group were mixed and treated as one sample. To eliminate experimental error, honey samples were harvested from the same comb at different stages of the in-hive ripening process. Sampling was carried out at 1 day when the comb cell was full of nectar, and 5, 10, and 15 days later, with the sampled honey referred to as 5 day-honey, 10 day-honey and 15 day-honey. Honey was collected with a sterilized spatula, placed directly into sterile tubes and was stored at 4°C until use. The vitex flowers were collected directly from plants close to the bee hives and stored in sterile tubes. The DNA extraction was done as soon as possible when the honey samples arrived at lab. The storage time of honey samples at 4°C was less than 24 h.

### Determination of Moisture Content and Phenolic Compounds in Honey Samples

The determination of moisture was ascertained by refractometry using an Abbe refractometer (Digital refractometer Atago, Germany). All measurements were performed at 40°C after waiting for 6 min for samples to equilibrate. The corresponding % moisture (g/100 g honey) was determined from the refractive index of the honey sample by consulting a standard table (AOAC, 1990).

Total of 16 phenolic compounds including gallic acid (GA), protocatechuic, *p*-hydroxybenzoic acid, caffeic acid, *p*-coumaric acid, ferulic acid, benzoic acid, rutin, quercetin, naringenin, kaempferol, apigenin, pinocembrine, caffeic acid phenethylester, chrysin and galangin were determined by HPLC-ESI-MS/MS according to our previously published method (Zhou et al., 2014; Wen et al., 2017).

### DNA Isolation, PCR Amplification, Amplicon Quantification and Pyrosequencing

The vitex flowers were surface sterilized with 75% ethanol for 1 min, followed by 30 s in 1% sodium hypochlorite solution and washed in sterile distilled water for 1 min, and ground into small pieces. Microbial genomic DNA was isolated using the Powersoil DNA isolation kit (MoBio Laboratories, Carlsbad, CA, United States) according to the manufacturer’s instructions, with some modification.10.0 g honey samples were suspended in 50 ml sterilized ddH2O, the solution was filtered through sterilized 0.22 μm pore-size filter membrane (Millipore Filter Membrane, Organic-system, 0.22 μm/50 mm, United States) to collected the microbial cells, the filter membranes were sterilized for three times before use and filtration was conducted in sterilized conditions. After filtration, the membrane with microbial cells were cut to small pieces and extracted by the Powersoil DNA isolation kit. All DNA was stored at -20°C prior to analysis. The V3-V4 regions of the 16S rRNA gene and the ITS rDNA region were subjected to high-throughput sequencing by Beijing Auwigene Tech, Ltd. (Beijing, China) using the Illumina Miseq PE300 sequencing platform (Illumina, Inc., San Diego, CA, United States).

PCR amplification of the V3-V4 region of the bacterial 16S rRNA gene was performed using the universal primers 336F (5′-GTACTCCTACGGGAGGCAGCA-3′) and 806R (5′-GTGGACTACHVGGGTWTCTAAT-3′) with incorporated sample barcode sequences. PCR amplification of the fungus ITS1 rDNA region was performed using the primers ITS1-F (5′-CTTGGTCATTTAGAGGAAGTAA-3′) and ITS1-R (5′-TGCGTTCTTCATCGATGC-3′) with incorporated sample barcode sequences. The PCR condition used were as follows: 5 min initial denaturation at 95°C; 25 cycles of denaturation at 95°C (30 s), annealing at 56°C (30 s), elongation at 72°C (40 s); and final extension at 72°C for 10 min. The PCR products were separated by 1% agarose gel electrophoresis and the approximately 460 bp band was purified by using the Agencourt AMPure XP kit (Beckman Coulter, Inc., CA, United States). Sequencing was performed using the Illumina Miseq PE300 sequencing platform (Illumina, Inc., San Diego, CA, United States) according to the manufacturer’s recommendations. The sequencing data was deposited in the NCBI Sequence Read Archive (SRA) sequence database with accession number SRP111523.

### Bioinformatics Analyses

Quality filtering and clustering was performed by a customized pipeline based on Uparse (Edgar, 2013; Edgar and Flyvbjerg, 2015) implemented in Usearch v.8 (Edgar, 2010), with some modifications. Paired-end reads were merged using the Usearch fastq mergepairs algorithm (Edgar and Flyvbjerg, 2015), allowing staggered alignment constructs in order to accommodate potentially short ITS1 and 16s rDNA amplicons. Reads not matching the primers or having read lengths below 300 (16S V3-V4) or 100 bp (ITS1) were discarded. Trimmed reads were quality-filtered using the Usearch fastq filter function with a maximum expected error threshold of one, and singletons were removed to obtain the final quality results. Sequences were clustered into OTUs at the 97% sequence identity using the Usearch cluster OTU function that includes an “on-the-fly” chimera detection algorithm (Edgar, 2013). Prokaryotic sequences identified as originating from organelles (chloroplasts, mitochondria), as well as eukaryotic sequences identified as originating from soil microbes metazoans, protists or plants (viridiplantae), or that were of an unknown eukaryotic origin, were removed from downstream analysis. Taxonomic classification of the representative sequence for each prokaryotic OTU was performed using Silva (Release 119^1^) and NCBI (Pruesse et al., 2007), and fungal OTUs were classified using the fungal ITS database, Unite (Release 7.0^2^) and NCBI (Kõljalg et al., 2005). After obtaining draft OTUs, singletons were removed to obtain the final quality results.

### Data Analyses

Data analysis was conducted by using the packages vegan with the statistical plantform R. Non-metric multidimensional scaling (NMDS) were used to test the differences in microbial community composition among different honey samples, using the meta MDS in “Vegan” package of R. Mothur was used to estimate the a and β diversity of bacterial and fungal. Before diversity analysis, the data of 15 samples in five groups were to normalize the abundance to the equivalent of 51868 reads for fungi and 5175 reads for bacteria. For α diversity analysis, Chao 1 index and the abundance-based coverage estimator (Ace) index were calculated by Mothur, and the Shannon-Winner index and Simpson index were calculated by the R package “vegan” to estimate the bacterial and fungal community richness within each sample. In β diversity analysis, Bray-Curtis dissimilarity matrixes for bacterial and fungal communities were constructed on the log_2_-transformed OTU relative abundance. OTUs with a relative abundance above 0.5% in at least one sample were included in the analysis. Rarefaction curves were performed to evaluate the sufficiency of the sampling effort within the software “Mothur.” Canonical correspondence analysis (CCA) was performed to measure chemical properties that have the most significant influence on microbial communities. CCA analysis was completed with *cca* in R with a stepwise model from the vegan package v2.4.3 (Oksanen et al., 2011). The significate difference of chemical and physical characteristics and relative abundances of different genera in each sample were performed by one factor ANONVA in SPSS version 21.0(SPSS Inc, Chicago, IL, United States). The core microbiota was defined as the microbial genera with more than 1% of total relative abundance among samples.

### General Characterization of Sequence Data

After quality trimming, a total of 632,225 high-quality sequence reads of 16s rDNA were acquired and the average reads per sample was 42,148 sequences. The sequences were assigned to a total of 405 bacterial OTUs at 97% sequence similarity after removing sequences belonging to plants, chimeras and denoising. After blasting in the NCBI and Silva databases, there were 14 phyla, 23 classes, 44 orders, 82 families and 160 genera of bacteria identified. The number of high-quality sequence reads obtained from ITS1 rDNA Mi-Seq was 1,051,844, and the average reads per sample was 70,122 sequences. The sequences were assigned to a total of 333 eukaryotic operational taxonomic units (OTU) at 97% sequence similarity, and among them, 320 OTUs were found to belong to fungi after depositing the low-quality sequences, chimera and denoising. After blasting in the NCBI and UNITE databases, there were 9 phyla, 23 classes, 49 orders, 72 families, and 138 genera of fungi. The taxa and abundance of bacterial OTUs and fungal OTUs across all samples are summarized in **Supplementary Tables S1, S2**.

Rarefaction curves (**Supplementary Figures S1A,B**) showed that most bacterial and fungal diversity was recovered and a sufficient sampling depth was attained for a complete understanding of diversity of microbiota across all samples. The diversity indices for total bacteria and fungi in different samples are shown in **Table 1**. The Shannon–Weiner and Simpson index indicated that the bacterial community diversity was not significantly different in the 1, 5, 10, and 15 day-honey sample and flower at the 3% cutoff values (*P* < 0.05). The richness indices for Chao1 and ACE indicated that the richness of the bacterial community showed no significant difference between the five samples (*P* < 0.05). With regards to the fungal community, the flower sample had the highest level of diversity, followed by the1 day-honey sample, the 5, 10, and 15 day-honey showed no significantly difference. And the community richness exhibited no significant difference among the five samples.

**Table 1:** Richness and diversity indices of total bacterial and fungal communities.

	Bacteria					Fungal				
Sample	Shannon	Simpson	Ace	Chao1	Coverage	Shannon	Simpson	Ace	Chao1	Coverage
Flower	1.47 ± 0.26a	0.39 ± 0.07a	209.9 ± 84.8a	147.6 ± 51.4a	0.99a	2.03 ± 0.17a	0.21 ± 0.05a	225.2 ± 28.7a	207.1 ± 7.1a	0.999a
1 day	1.68 ± 0.08a	0.36 ± 0.02a	272.6 ± 65.1a	181.4 ± 49.8a	0.99a	1.69 ± 0.06b	0.38 ± 0.01b	222.0 ± 9.3a	216.2 ± 9.2a	0.999a
5 days	1.59 ± 0.03a	0.38 ± 0.00a	187.8 ± 107.9a	131.1 ± 59.9a	0.99a	0.66 ± 0.14c	0.79 ± 0.04c	196.8 ± 47.7a	175.0 ± 51.0a	0.999a
10 days	1.70 ± 0.06a	0.35 ± 0.01a	212.5 ± 60.5a	159.9 ± 49.9a	0.99a	0.57 ± 0.09c	0.82 ± 0.03c	246.6 ± 47.5a	205.1 ± 23.2a	0.999a
15 days	1.71 ± 0.07a	0.36 ± 0.02a	198.6 ± 37.2a	152.3 ± 31.4a	0.99a	0.60 ± 0.18c	0.80 ± 0.06c	228.3 ± 62.5a	171.0 ± 22.1a	0.999a

*Chao 1 and Ace indexes were used to evaluate the community richness, while Shannon and Simpson indexes were used to assess the community diversity. Different letters represent significant differences between honey samples and flower.*

### Bacterial and Fungal Community Composition in Flowers and Honeys with Different Ripening Times

**FIGURE 1:**
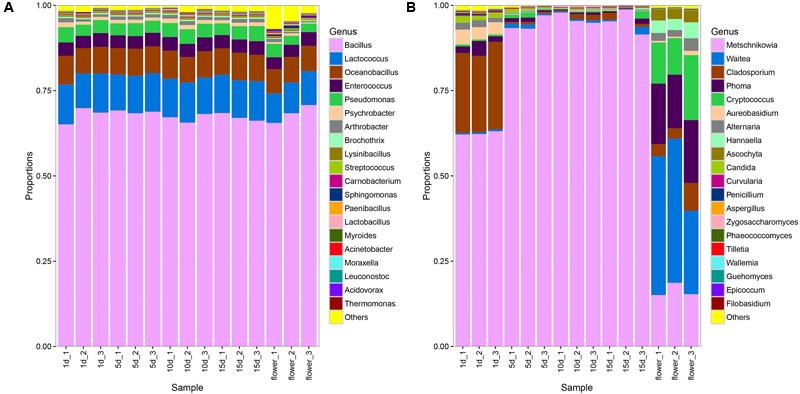
Bacterial and fungal community patterns in different honey samples. **(A)** Bacterial community at the genus level, **(B)** fungal community at the genus level. The percentages along the vertical axis of the graph represent the proportion of each microbe group in the total obtained sequences from each sample.

A total of 164 bacterial genera were detected in vitex flowers and honeys. The composition of bacterial community in the vitex flowers and honey was quite similarly, while the proportion varied among honey samples and flower (**Figure 1A**). The bacterial profiles of vitex flowers were dominated by *Bacillus* spp., *Lactococcus* spp., *Oceanobacillus* spp., *Enterococcus* spp., *Pseudomonas* spp. with an average relative abundance of 68, 9, 7, 3, and 3%, respectively. Similar with the vitex flowers, bacterial profiles of honey samples predominantly consisted of the *Bacillus* spp., *Lactococcus* spp., *Oceanobacillus* spp., *Enterococcus* spp., *Pseudomonas* spp., *Psychrobacter* spp., and *Arthrobacter* spp., with 67, 11, 7, 4, 4, 1, and 1 relative abundances, respectively. There were no significant differences with the bacterial community composition in honey at different ripening stages (*P* > 0.05; **Supplementary Table S3**).

Six OTUs (OTU 1, 3, 12, 18, 31, and 62) were assigned to the *Bacillus* genus (**Table 2**). These OTUs were recovered in all honey samples and the vitex flowers. OTUs 1 and 3 accounted for 97.4–98.2% of the *Bacillus*-related sequences in the honey and flower samples. These OUT related sequences were frequently found in soil and environmental samples in previous studies (Logan and Vos, 2015).

**Table 2:** Relative abundance of main bacterial OTUs at genus level in honey sample.

Related genera	Related species	OTU-id	Relative abundance	Accession number	*e*-value	Similarity
*Bacillus*	*Bacillales* AT06-06	OTU_12	0.53%	KM349937.1	1.00E-125	100
	*Bacillus badius*	OTU_62	0.02%	KJ452465.1	1.00E-125	100
	*Bacillus xiamenensis*	OTU_31	0.95%	KM817225.1	1.00E-125	100
	*Bacillus* sp. N5/665	OTU_1	52.36%	LN680102.1	1.00E-125	100
	*Bacillus flexus*	OTU_3	13.18%	KP261065.1	1.00E-125	100
	*Bacillus firmus*	OTU_18	0.16%	LC019792.1	1.00E-125	100
*Lactococcus*	*Lactococcus lactis*	OTU_26	2.28%	KP064393.1	1.00E-123	99.6
	*Lactococcus piscium*	OTU_4	8.98%	JN226415.1	3.00E-125	100
*Oceanobacillus*	*Oceanobacillus* sp. HB12159	OTU_5	7.85%	KJ423095.1	1.00E-125	100
*Enterococcus*	*Enterococcus* sp. AfA50	OTU_7	3.95%	KM586976.1	1.00E-125	100
*Pseudomonas*	*Pseudomonas* sp. VT1B	OTU_49	0.06%	AB819626.1	2.00E-110	95.67
	*Pseudomonas* sp. NCCP-645	OTU_8	2.46%	AB920823.1	1.00E-125	100
	*Pseudomonas mendocina*	OTU_39	0.06%	KP165490.1	1.00E-125	100
	*Pseudomonas* sp. NCCP-624	OTU_11	1.19%	AB920809.1	1.00E-125	100
*Psychrobacter*	*Psychrobacter maritimus*	OTU_57	0.05%	KF424829.1	1.00E-125	100
	*Psychrobacter cibarius*	OTU_9	0.94%	KF600781.1	1.00E-125	100
	*Psychrobacter* sp. ST4(2013)	OTU_29	0.03%	KF013871.1	1.00E-125	100
*Arthrobacter*	*Arthrobacter phenanthrenivorans strain PB-3*	OTU_10	0.99%	KP257601.1	1.00E-125	100

*The identification of bacterial species was conducted by blast sequence with NCBI database.*

Two OTUs were assigned to the genus *Lactococcus*, accounting for 11.0–11.2% of the bacterial community in the honey sample. The genus OTU 26 (accounting for 1.9–2.3% of the bacterial community) showed a high 16s rRNA gene sequence similarity (99.6%) to *Lactococcus lactis* strain A5 (KP064393.1); *L. lactis* has been used for decades by the dairy industry. OTU 4 (accounting for 8.9–9.3% of the bacterial community) exactly matched the 16S rRNA gene sequence of *Lactococcus piscium* strain MARL41 (JN226415.1). Interestingly, a previous study showed that *L. piscium* strains could be isolated from seafood and packaged meat (Rahkila et al., 2012). *Lactococcus* was detected in the gut of *A. cerana* honey bees (Ahn et al., 2012), and *Lactococcus* has not been traditionally considered as spoilage organisms.

In agreement with a previous report, *Enterococci* and *Pseudomonas* sp. were detected in the vitex honey (Snowdon and Cliver, 1996; Olaitan et al., 2007). *Pseudomonas* and *Enterococcus* are environmental genera (Corby-Harris et al., 2014) and had stable communities during honey ripening. The *Psychrobacter* and *Arthrobacter* genera had stable communities during the honey ripening process, and thus was not influenced by the honey ripening.

In addition, the *Oceanobacillus*-related OTU 5 accounted for a stable proportion (7.8%) of the bacterial community of the honey, also was the third dominate bacteria (7.4%) in flower, indicating that the *Oceanobacillus* in honey probably from flower-origin, and could survival in sugar environment of honey. Members of the genus *Oceanobacillus* are aerobic, halophilic bacteria widely distributed in various environments such as marine environments (Kim et al., 2015), soil (Lee et al., 2013; Wu et al., 2014), fermented foods (Namwong et al., 2009; Tominaga et al., 2009).

With regards to the ITS sequence, the fungal community of vitex flowers and honey samples were heterogeneous (**Figure 1B**). The fungal profiles of vitex flowers predominantly consisted of *Waitea, Phoma, Metschnikowia, Cryptococcus*., *Cladosporium, Hannaella, Ascochyta*, and *Alternaria*, representing 36, 17, 16, 14, 4.9, 3.9, 3.2, and 2.6% of the total identified genera, respectively. In the honey samples, *Metschnikowia, Cladosporium, Aureobasidium, Alternaria, Phoma*, and *Candida* were identified as dominate genera and found at greater than 1% of average relative abundance across the twelve samples. *Metschnikowia* and *Cladosporium* were dominate in ripen honey and had an average relative abundance of 96 and 1%, respectively. While, *Aureobasidium* (3.8%), *Phoma* (2.7%), *Alternaria* (1.8%), and *Candida* (1.5%) were additional core microbiota in 1 day-honey. The other fungal species accounting for only a small proportion (<1%) (**Figure 1B**). The fungal community structure showed significant differences between the 1 days-honey sample and the 5, 10, and 15 day-honey samples. Statistically, the relative abundance of *Metschnikowia* genus were found to be significantly higher in 5 day-honey than 1 day-honey samples (*P* < 0.05, **Supplementary Table S4**), the proportion of *Metschnikowia* tended to be stable and did not change significantly over the course of the 5, 10, and 15 days sampling times. whereas another five genus: *Cladosporium, Aureobasidium, Alternaria Phoma*, and *Candida* were significantly lower in 15 day-honey than 1 day-honey samples (*P* < 0.05, **Supplementary Table S4**).

It is notable that *Waitea, Cryptococcus* and *Hannaella* was the dominant species observed in the vitex flower, while the proportion of these three genus declined to be only a small proportion (<1%) of the fungal community in honey.

### Comparison of Microbial Communities in Vitex Flowers and Honeys with Different Ripening Times

**FIGURE 2:**
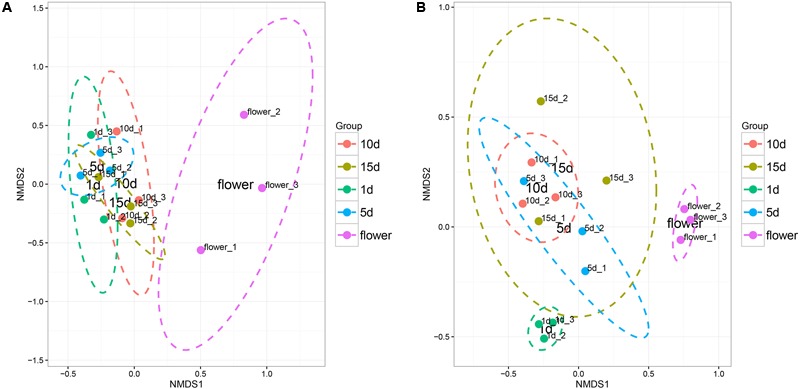
Non-metric multidimensional scaling (NMDS) ordination of bacterial **(A)** and fungal **(B)** community structure inferred from the relative abundance of OTUs. NMDS analysis (Stress value: 0.08). Ellipses indicate confidence intervals of 90%.

The Non-metric dimension scaling (NMDS) ordination (**Figure 2**) of the bacterial communities in the flower and honey assemblages showed a broad split between the two groups. All the honey samples ripened for different times showed similar bacterial composition with little temporal dynamics. ANOVA tests showed that the relative abundances of bacterial community was no significantly difference among different repining honey samples (Tukey Test, *P* > 0.05, **Supplementary Table S3**). NMDS plots were sufficient for visualizing the separation of flowers from honeys (**Figure 2A**). NMDS plots were insufficient to distinguish the 1 day-honey from the 5 day-honey or the 10 day-honey from the 15 day-honey. Compared to bacterial communities, NMDS ordination of the fungal communities formed three clusters, which successfully showed separation of the vitex flower and 1 day-honey samples from the 5, 10, and 15 day-honeys (**Figure 2B**). This result suggests that the fungal community during the initial stages of ripening is different from the ones in the latter stages, whereas the microbial community did not change a great deal. Fungal diversity, as determined by the Shannon index and Simpson reciprocal index, did not show significant differences among the samples.

**FIGURE 3:**
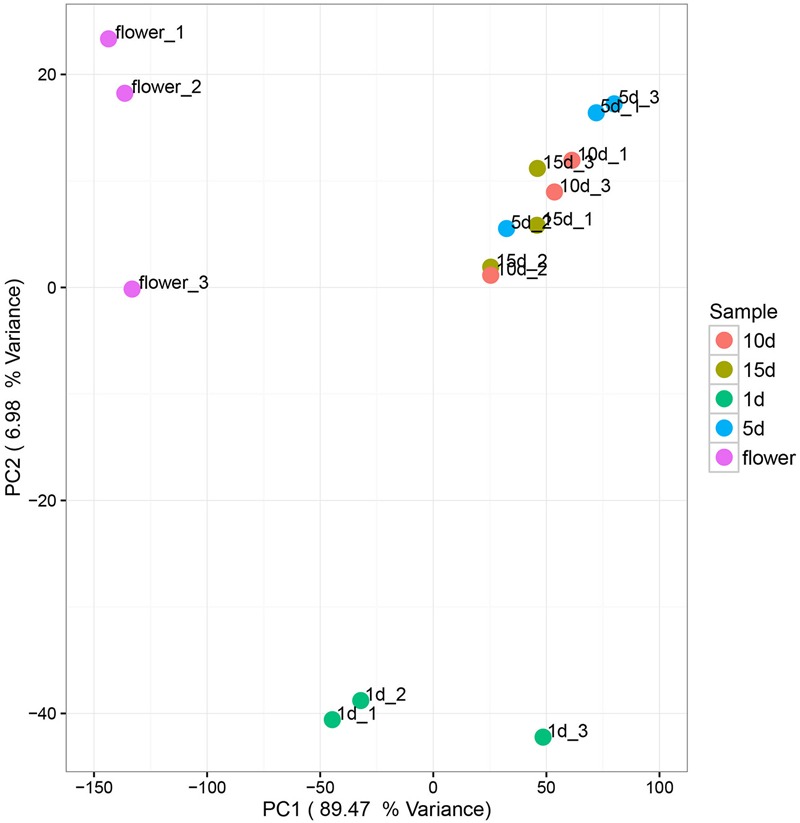
Principal component analysis of fungal communities in flowers and different honey samples. Fungal community relationships between the 15 samples, percentage values along the axes of the graph represent the explained variance of the total variance.

Principal components analysis (PCA) was used to analyze the relationship between the microbial communities in the flower and honey samples. According to the PCA results, the fungal communities were different between the five samples. The first two Principal Components (PCs) explained 96.45% of the total variance (**Figure 3**). Along the first and second component, the isolates were clearly grouped into three clusters, suggesting that isolates belonging into the same cluster share relevant characteristics. The vitex flower and 1 day-honey were separated from the 5, 10, and 15 day-honeys based on the fungal communities, consistent with the NMDS results. However, PCA ordination of flowers and honey samples based on bacterial communities were irregular, and the results were not included.

### Relationships between Honey Chemical Variables and the Microbial Community Structure

Phenolic and water content in honey samples was listed in **Table 3**. Water content showed a significantly decreased trend along with the honey ripening, which was consistent with the previous research (Ruiz-Argueso and Rodriguez-Navarro, 1975). Total of 16 phenolic compounds were detected in vitex honey, including GA, protocatechuic, *p*-hydroxybenzoic acid, caffeic acid, *p*-coumaric acid, ferulic acid, benzoic acid, rutin, quercetin, naringenin, kaempferol, apigenin, pinocembrine, caffeic acid phenethylester, chrysin and galangin. Most of the phenolic compounds showed an first increased and then decreased trend during the honey ripening process, for example, protocatechuic, caffeic acid, *p*-coumaric acid, benzoic acid, rutin, naringenin, chrysin and galangin. The content of kaempferol and apigenin sharply decreased to relatively low level along with the honey ripening. GA and *p*-hydroxybenzoic acid showed an increased trend along with the honey ripening. *p*-hydroxybenzoic acid, *p*-coumaric acid, GA and protocatechuic showed relatively high level in the repining honey.

**Table 3:** Phenolic and water content in honey samples at different ripening stage.

Compounds	Abbreviation	Sample			
		1 day	5 days	10 days	15 days
Water content	water	31.85 ± 0.94a	22.27 ± 0.80b	19.23 ± 0.11c	18.58 ± 0.36c
Gallic acid	GA	3.18 ± 0.15d	9.36 ± 0.44c	25.89 ± 0.93b	21.66 ± 0.52a
Protocatechuic	PR	8.62 ± 0.08c	14.56 ± 1.65b	20.55 ± 1.31a	12.75 ± 1.08b
*p*-Hydroxybenzoic acid	PHA	183.40 ± 2.01c	187.31 ± 1.73b	232.39 ± 1.03a	230.25 ± 1.02a
Caffeic acid	CA	42.82 ± 1.73c	76.18 ± 1.30c	106.52 ± 0.57a	41.24 ± 0.95b
*p*-Coumaric acid	PCA	33.34 ± 1.25c	45.42 ± 1.06b	49.79 ± 1.10a	33.58 ± 0.89c
Ferulic	FE	11.91 ± 0.68b	13.58 ± 0.54a	11.55 ± 0.62b	6.20 ± 0.26c
Benzoic acid	BA	1.55 ± 0.30c	2.28 ± 0.13b	5.26 ± 0.17a	2.02 ± 0.20b
Rutin	RU	0.84 ± 0.12d	2.31 ± 0.14b	5.08 ± 0.17a	1.80 ± 0.15c
Quercetin	QU	0.06 ± 0.01b	0.10 ± 0.01a	0.08 ± 0.01b	0.07 ± 0.01b
Naringenin	NAR	2.75 ± 0.12d	4.62 ± 0.15a	4.22 ± 0.12b	2.99 ± 0.05c
Kaempferol	KA	1.81 ± 0.10a	1.01 ± 0.10b	1.13 ± 0.03b	0.75 ± 0.09c
Apigenin	AP	4.77 ± 0.14a	0.77 ± 0.06b	0.62 ± 0.10b	0.55 ± 0.11b
Pinocembrine	PI	9.74 ± 0.34d	14.60 ± 0.30a	12.45 ± 0.28b	10.37 ± 0.27c
Caffeic Acid Phenethylester	CAP	7.41 ± 0.25c	8.51 ± 0.36a	5.70 ± 0.19d	7.97 ± 0.15b
Chrysin	CH	4.04 ± 0.14d	26.19 ± 0.18a	15.95 ± 0.38b	9.03 ± 0.28c
Galangin	GAL	0.85 ± 0.12c	3.33 ± 0.20b	4.27 ± 0.17a	0.63 ± 0.08c

**FIGURE 4:**
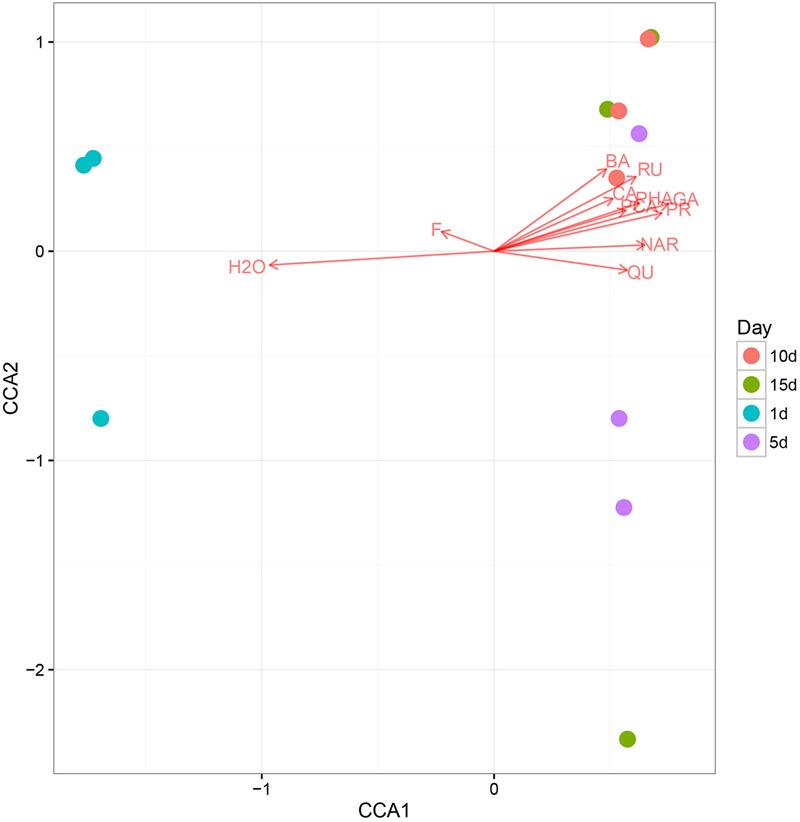
The canonical correspondence analysis (CCA) loading plot of the fungal structure composition in relation to honey chemical properties. Chemical properties: H_2_O, moisture; GA, gallic acid; PR, protocatechuic; PHA, *p*-Hydroxybenzoic acid; CA, caffeic acid; PCA, *p*-coumaric acid; FE, ferulic acid; BA, benzoic acid; RU, rutin; QU, quercetin; NAR, naringenin; KA, kaempferol; AP, apigenin; PI, pinocembrine; CAP, caffeic acid phenethylester; CH, chrysin; GAL, galangin.

Using CCA, we identified honey properties with the largest effect on the honey fungal community structure at each of the sampling sites (**Figure 4**). The moisture content had the smallest effect on CAA2 among the studied variables but had the largest effect on CAA1. The CCA biplot showed that 1 d-honey samples were located in a negative direction from CAA1, thus, these organisms were related positively to honey moisture (H_2_O) and ferulic acid (FA). The other honey samples were grouped in a positive direction along CCA1, and the 10 day-honey and 15 day-honey samples grouped closely in the positive direction of CCA2, indicating that the fungal community in these samples were related to the phenolic compounds, for example, GA, protocatechuic acid (PR) and *p*-hydroxybenzoic acid (PHA). The CCA analysis of the bacterial community data was not listed, since the results did not show a typical correlation with the honey ripening patterns.

## Discussion

Honey is the result of the transformation of nectar from plants by honey-bees. Nature ripening process was complexity and necessary for honey produce. Due to the natural properties of honey and control measures in the honey industry, honey is a product with minimal types and levels of microbes. However, microbiological index was associated with honey quality and safety, and for this purpose, a better comprehensive understanding of its microbiological characteristics has to be achieved. The microbiota investigation of this study by sequencing technology provided a comprehensive understanding of the microbiome community changes during the honey repining.

### Bacterial Community Structure

Based on the 16S rRNA gene analysis, most of the reads from the microbial habitats could be sorted into known phyla. At the genus level, in total, 98.5∼99.5% of the bacterial sequences covering the V3–V4 regions were given taxonomical assignments.

The primary sources of microbial in honey are likely to include pollen, the digestive tracts of honeybees, dust, air and flowers. It has been suggested that flowers and hives are more important sources of microbes than the soil (Root, 1993). While, *Bacillus, Enterococcus*, and *Pseudomonas* were also found in digestive tract of honey bee (Kacániová et al., 2009; Engel et al., 2012); and *Lactococcus* was detected in the gut of *A. cerana* honey bees (Ahn et al., 2012). In our study, the first five predominant bacterial communities in the honey samples and flower including *Bacillus, Lactococcus, Oceanobacillus, Enterococcus*, and *Pseudomonas* were the same. We further confirmed the results from others that bacteria prevalent in honey has the transmission with the nectar and also floral (Anderson et al., 2013).

Most bacteria cannot grow or reproduce in honey, i.e., they are dormant and this is due to antibacterial activity of honey. *Bacillus* spp. are most prevalent bacterial in honey. Our results showed that *Bacillus* constituted more than 67% of bacterial of either 1 day honey or ripening honey, which was in agreement with previous work (Iurlina and Fritz, 2005; Alippi and Reynaldi, 2006). Most identified *Bacillus* species are considered safe except for two (*Bacillus anthracis* and many *B. cereus* toxin-producer strains). Some studies have indicated that *Bacillus* species are tolerant to abiotic stresses. Several *Bacillus* strains have the ability to produce antibiotics, bacteriocins, or antifungal compounds that have been used for agricultural and healthcare purposes (Duc et al., 2004; Alfonzo et al., 2012). Interestingly, *Lactococcus* and *Oceanobacillus* were detected in honey for the first time by pyrosequencing analysis; according to previous study, *Lactococcus* was detected in the gut of honey bees (Ahn et al., 2012); *Oceanobacillus* related species generally was isolated from marine environments and could survived in hypertonic environment(Lee et al., 2013; Kim et al., 2015). Our findings here suggested that *Oceanobacillus* spp. may survival in honey stress environment.

### Fungal Community Structure

Yeast was the dominate fungi in honey identified used culture- and molecular-based methods, such as *Zygosaccharomyces, Debaryomyces*, and *Candida*, have been identified in honey previously (Sinacori et al., 2014). In our study, Two predominant OTU1 and OTU42 occupied 92% of the total OTUs abundance, was closely related to the *Metschnikowia pulcherrima* strain S1740 (82.72 and 84.71%) of the family *Ascomycota*, thus these two OTUs were assigned to the genus of *Metschnikowia*. Totally, 14 OTUs were assigned to the *Metschnikowia* (**Table 4**). The *Metschnikowia* of yeast was found to be extremely dominant in the honey samples. This is the first report of *Metschnikowia* yeast being detected in honey, which could have been the result of differences in the environment in which the bee farm was located. *Metschnikowia* species were also present at relatively high levels in the vitex flower, indicating it to be the source of *Metschnikowia* in the honey. Previous studies indicated that *Metschnikowia* spp. were a dominant yeast community in nectar and could be easily isolated from flowers or fruits and transmitted to new niches by insects (Hong et al., 2001; Lievens et al., 2015). One study found that *M. pulcherrima* displayed a broad and effective antimicrobial action on undesired wild spoilage yeasts, such as those of the genera of *Brettanomyces, Dekkera, Hanseniaspora*, and *Pichia* (Oro et al., 2014).

**Table 4:** Relative abundance of main fungal OTUs at genus level in honey sample.

Related genus	OUT-id	Related species	Relative abundance %	*e*-value	Similarity
*Metschnikowia*	OTU_1	*Metschnikowia pulcherrima*	89.4	9.00E-35	82.72
	OTU_42	*Metschnikowia pulcherrima*	3.9	2.00E-37	84.71
	OTU_258	*Metschnikowia pulcherrima*	0.6	3.00E-34	83.54
	OTU_92	*Metschnikowia pulcherrima*	0.2	1.00E-33	82.1
	OTU_259	*Metschnikowia pulcherrima*	0.2	5.00E-32	80.61
	OTU_184	*Metschnikowia pulcherrima*	0.2	3.00E-34	83.02
	OTU_24	*Metschnikowia pulcherrima*	0.2	7.00E-36	83.75
	OTU_143	*Metschnikowia pulcherrima*	<0.1	9.00E-35	83.12
	OTU_317	*Metschnikowia pulcherrima*	<0.1	5.00E-31	81.88
	OTU_116	*Metschnikowia pulcherrima*	<0.1	7.00E-22	93
	OTU_127	*Metschnikowia pulcherrima*	<0.1	6.00E-31	81.25
	OTU_108	*Metschnikowia pulcherrima*	<0.1	3.00E-28	79.04
	OTU_252	*Metschnikowia pulcherrima*	<0.1	1.00E-33	83.02
	OTU_325	*Metschnikowia pulcherrima*	<0.1	2.00E-31	80.86
	OTU_255	*Metschnikowia pulcherrima*	0.1	4.00E-32	81.88
	OTU_254	*Metschnikowia pulcherrima*	<0.1	2.00E-30	84.03
*Cladosporium*	OTU_6	*Cladosporium cladosporioides*	0.2	3.00E-113	100
	OTU_10	*Cladosporium cucumerinum*	0.1	5.00E-116	100
	OTU_74	*Cladosporium* sp. MBC003	<0.1	6.00E-109	98.28
	OTU_5	*Cladosporium* sp. N62	0.7	8.00E-114	100
*Phoma*	OTU_4	*Phoma* sp. strain G10	0.7	6.00E-74	100
	OTU_129	*Phoma* sp. BPL2_2	<0.1	2.00E-95	97.7
	OTU_56	*Phoma* sp. CY107	<0.1	3.00E-105	100
*Alternaria*	OTU_39	*Alternaria brassicae*	<0.1	3.00E-119	100
	OTU_18	*Alternaria* sp. BRO-2013	<0.1	6.00E-135	100
	OTU_210	*Alternaria* sp. Cs36-5	<0.1	5.00E-46	99
	OTU_7	*Alternaria* sp. G57	0.2	4.00E-118	100
*Aureobasidium*	OTU_8	*Aureobasidium pullulans*	0.1	3.00E-126	100
*Candida*	OTU_19	*Candida rugosa*	<0.1	1.00E-63	100
	OTU_12	*Candida sake*	0.1	1.00E-76	99.4
	OTU_15	*Candida akabanensis*	<0.1	1.00E-64	100

Filamentous fungi, including those of the *Cladosporium, Alternaria*, and *Aspergillus*, are considered common contaminants of honey (Laila, 2004; Kacániová et al., 2009), were also found in the ripen honey samples. And *Cladosporium* and *Alternaria* related OTUs were also enriched in vitex flower. *Waitea* and *Hannaella* related OTUs was enriched in flowers, while showed lowly abundance in honey samples. According to previous research, *Waitea* and *Hannaella* related species was mostly isolated from plant and soil (Zhang et al., 2014; Kaewwichian et al., 2015), these species weren’t adapt to honey hyperosmosis environment.

### Correlation between Honey Ripening and Microbial Community

The ripening of nectar into honey occurs by a combination of two processes: the conversion of sucrose into glucose and fructose, and the evaporation of excess water. Our results showed that the water content showed a sharp decrement at 5 days ripening point, and decreased to less than 20% in honey after 10 days of ripening. Previous study suggested that the growth of any species and fermentation can occur in ripen honey if the water content is below 17.1% (Olaitan et al., 2007). Interestingly, in our study, the relative abundance of the *Cladosporium, Alternaria, Aspergillus*, and *Penicillium* genera sharply decreased in the 5 day-honey and was maintained at a low level. Mold fungi, such as those in the *Aureobasidium* and *Phoma* genera, also showed a sharp decrease in 5 day-honey. The antifungal action of honey has also been observed for some yeasts and species of *Aspergillus* and *Penicillium* (Brady et al., 1996). This may be related to the decrease of water content in honey during the ripening process. Honey that is harvested before it is completely ripe has a higher moisture content and may be vulnerable to spoilage. These results indicated that the management of honey ripening in hives was necessary.

It is known that high antimicrobial activity is as a result of osmotic effect, acidity, hydrogen peroxide and phytochemical factors. Due to the antibacterial properties of honey, most bacteria species was detected in low abundance, only some bacteria species were detected in relatively high level in the honey sample, which was consistent with previous researches (Olaitan et al., 2007; Kacániová et al., 2009). The most detected bacterial species community was stable during the honey ripening process, indicating that they are highly adapted to honey matrices. While the fungal community composition varied among the honey ripening process. And the CCA results showed that fungal community in the 10 and 15 day-honey was related to the decrease of moisture and the increase of some phenolic compounds during the ripening process (**Figure 4**). Phytochemical factors have been described as non-peroxide antibacterial factors, which are believed to be many complex phenols and organic acids (Olaitan et al., 2007; Estevinho et al., 2008). Estevinho’s results indicated that phenolic compounds in honey were partially responsible for the antibacterial properties of Northeast Portugal honey (Estevinho et al., 2008). Both these studies and our results suggest that phytochemical like phenolic more or less was related with the antifungal activity of ripening honey.

In summary, in ripen honey the most abundant bacterial genus was *Bacillus*, followed by *Lactococcus, Oceanobacillus, Enterococcus*, and *Pseudomonas*, and the fungal community was dominated by *Metschnikowia*, which was first reported in honey. Honey samples of different ripening stages exhibited no significant differences in bacterial composition. The fungal community composition was significantly different in honey from the 1 day samples compared to the 5, 10, and 15 days samples. The sharp decrease of relative abundance of filamentous fungi, mold fungi and candida yeast communities in ripen honey samples indicated that at least 5 days ripening period in the honeycomb was necessary for the honey production. The change of the fungal community was correlated with the variation in chemical components, such as moisture and phenolic compounds. The functional identification of core microbial species, as *Lactococcus* spp., *Oceanobacillus* spp., and *Metschnikowia* spp. would be the next targets for further researches.

## Author Contributions

Conceived and designed the experiments: LW, JZho, and YL. Performed the experiments: YW and LW. Analyzed the data: LS and XZ. Contributed reagents/materials/analysis tools: YJ and JZha. Wrote the paper: YW.

## Conflict of Interest Statement

The authors declare that the research was conducted in the absence of any commercial or financial relationships that could be construed as a potential conflict of interest.

## Footnotes

1 http://www.arb-silva.de

2 http://unite.ut.ee/index.php

## Supplementary Material

FIGURE S1 Rarefaction curve of bacterial **(A)** and fungal **(B)** OUT diversity for *vitex* flower and honey samples at different stages of ripening.

TABLE S1 Number of sequences in each of the bacteria OTUs found in *vitex* flowers and honey samples at different stages of ripening.

TABLE S2 Number of sequences in each of the fungal OTUs found in *vitex* flowers and different stage honey samples.

TABLE S3 *P*-value of ANOVA test of the top 30 bacterial genus among different ripening honey samples.

TABLE S4 *P*-value of ANOVA test of the top 30 fungal genus among different ripening honey samples.
