# Supplementary figures and images for: The Microbial Community Dynamics during the Vitex Honey Ripening Process in the Honeycomb
Source: Front Microbiol. 2017 Aug 29;8:1649. doi: 10.3389/fmicb.2017.01649 (PMC5583594; doi:10.3389/fmicb.2017.01649)

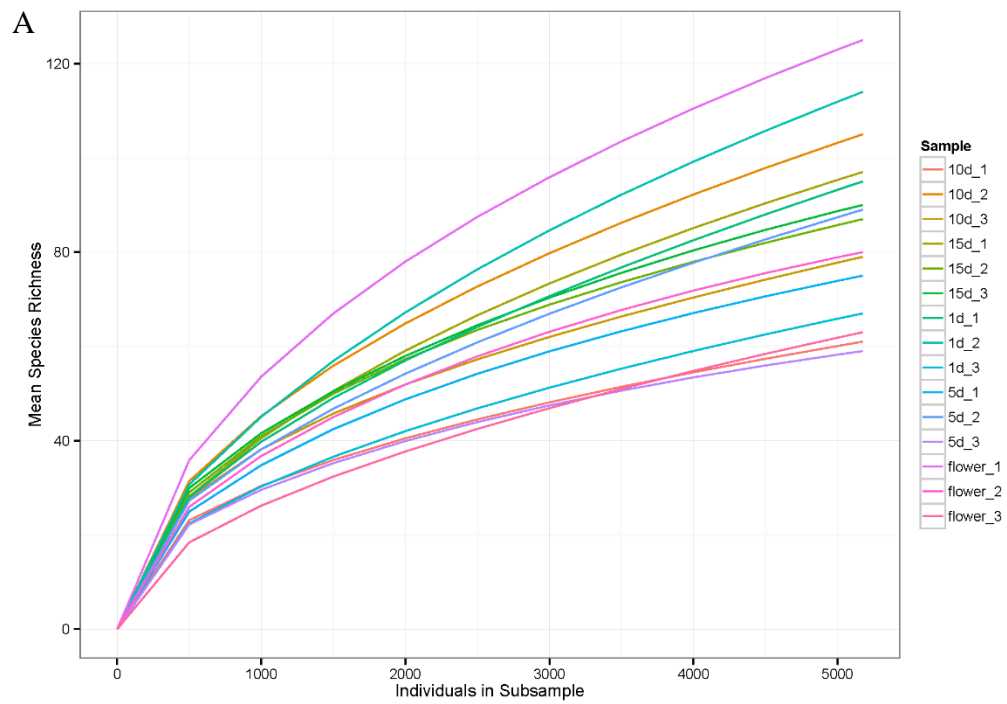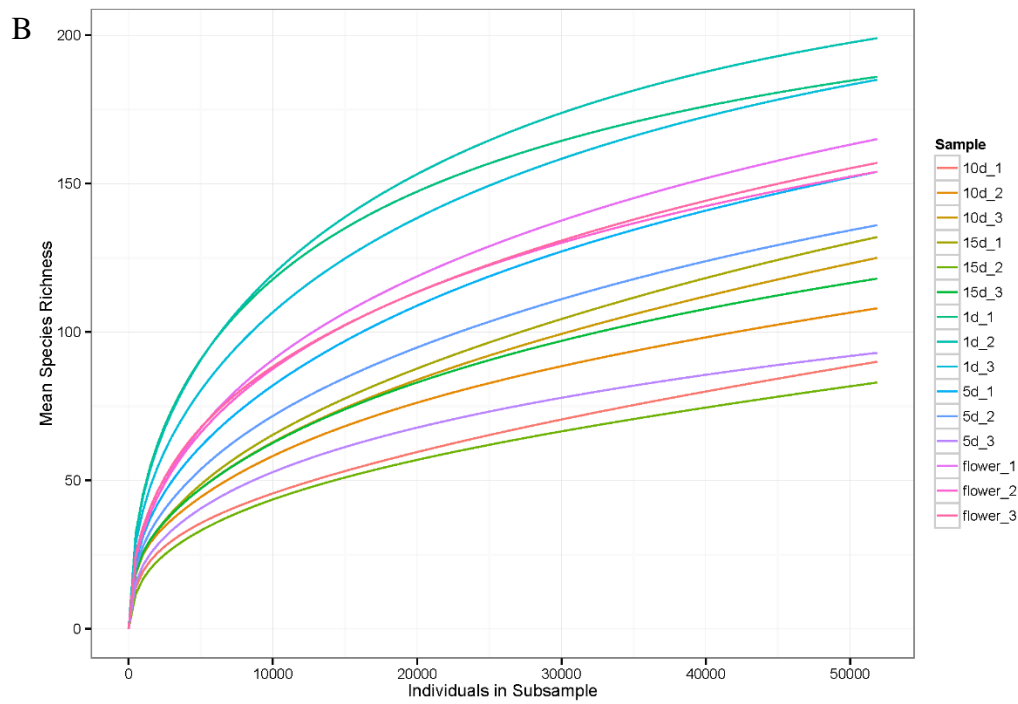

Supplement: FIGURE S1 — Rarefaction curve of bacterial (A) and fungal (B) OUT diversity for vitex flower and honey samples at different stages of ripening. [file Image_1.PDF]
